# QEEG indices in traumatic brain injury - insights from the CAPTAIN RTMS trial

**DOI:** 10.25122/jml-2024-0187

**Published:** 2024-03

**Authors:** Olivia Verişezan Roşu, Diana Chira, Vlad-Florin Chelaru, Diana Chertic Dăbală, Livia Livinț Popa, Ana-Maria Buruiană, Fior Dafin Mureşanu

**Affiliations:** 1 Department of Neurosciences, Iuliu Hațieganu University of Medicine and Pharmacy, Cluj-Napoca, Romania; 2 RoNeuro Institute for Neurological Research and Diagnostic, Cluj-Napoca, Romania; 3 Faculty of Medicine, Iuliu Hațieganu University of Medicine and Pharmacy, Cluj-Napoca, Romania; 4 Neurology Clinic, Cluj County Emergency Clinical Hospital, Cluj-Napoca, Romania

**Keywords:** TBI, qEEG, Cerebrolysin, rTMS, DAR, DTABR, TBR

## Abstract

This secondary analysis of the CAPTAIN-RTMS trial data focused on the significance of quantitative electroencephalography (qEEG) indices as indicators of recovery in patients with traumatic brain injury (TBI). By focusing on the delta alpha ratio (DAR), delta theta/alpha beta ratio (DTABR), and theta beta ratio (TBR), this study explored the shifts in brainwave activity as a response to an integrative treatment regimen of repetitive transcranial magnetic stimulation (rTMS) combined with the neurotrophic agent Cerebrolysin. Findings revealed significant increases in DAR and DTABR, suggesting changes in neurophysiological dynamics after treatment. However, variations in TBR were inconclusive in providing clear electrophysiological insights. These results indicate that further research is necessary to describe and understand the underlying mechanisms of brain recovery and to develop refined treatment frameworks for patients with TBI.

## INTRODUCTION

Traumatic brain injuries (TBI) are a major global health issue due to their widespread occurrence and consequential effects on individuals. TBI is characterized by a disturbance in brain function or other indications of brain pathology resulting from an external force [1]. The initial trauma triggers a cascade of events, including inflammation and oxidative stress, that can lead to further tissue damage and neurological complications. These secondary processes may involve a compromised blood-brain barrier, leading to brain swelling, increased pressure, and the release of excitatory neurotransmitters that can induce neuronal cell death. These multifaceted processes underscore the gravity of TBI, influencing patient outcomes and shaping the strategies employed for treatment [2].

### qEEG Parameters

Quantitative EEG (qEEG), a computer-assisted method for interpreting electroencephalographic recordings, reveals quantitative trends that may not be discernible through conventional visual analysis [3]. This approach has introduced novel techniques to extract features from EEG signals, such as analyzing specific frequency bands, signal complexity, and connectivity patterns within the brain network [4,5]. These analyses provide various parameters, such as the delta alpha ratio (DAR), delta theta/alpha beta ratio (DTABR), and theta beta ratio (TBR).

The delta alpha ratio (DAR) is a quantitative EEG measure computed by dividing the power in the delta frequency band by the power in the alpha frequency band. This ratio serves as a valuable indicator for assessing the equilibrium or imbalance between slow-wave (delta) and fast-wave (alpha) activity within the brain. Abnormal DAR values can provide insights into the neurophysiological dynamics associated with cognitive processes, contributing to a comprehensive understanding of brain function and potential implications for conditions affecting attention [6-9].

The delta theta/alpha beta ratio (DTABR) is derived by computing the ratio of delta and theta power to the combined power of alpha and beta waves. This ratio plays a crucial role in assessing the overarching balance between slower and faster frequency bands within the EEG. The interpretation of DTABR extends to the evaluation of cognitive functioning, where abnormalities in this ratio may indicate potential alterations in cognitive processes. Scientific evidence has linked abnormal DTABR values to various neurological conditions, emphasizing the significance of this measure in understanding the complexities of brain activity and its implications for cognitive health [6,10-13].

The theta beta ratio is calculated by dividing the power in the theta frequency band by the power in the beta frequency band. TBR has emerged as a significant metric in neurophysiological investigations, providing insights into the intricate dynamics of attention and cognitive performance. An abnormal TBR may signify challenges in attention, concentration, or overall cognitive functioning. The utilization of TBR as an indicator can offer a nuanced understanding of cortical activity, contributing to the identification and exploration of cognitive issues within diverse neurological contexts [14-16].

### Interventions for TBI recovery

Repetitive transcranial magnetic stimulation (rTMS) is a neuromodulation method that uses swiftly oscillating magnetic fields to activate neural functions. This process involves transmitting short electrical currents through a coil, generating a magnetic field that triggers cortical neurons situated beneath the focal area of the coil [17]. This technique can be adjusted to influence both local and distant brain regions by varying coil location and stimulation frequency. Typically delivered in repetitive pulses at consistent intervals, this technique displays varied effects based on frequency. High-frequency stimulation, 5 Hz or higher, is thought to heighten neuronal excitability, while low-frequency stimulation, under 1 Hz, is linked to inhibitory outcomes [18,19]. One notable benefit of rTMS is its remarkable safety record and absence of notable adverse effects [17].

Although rTMS has undergone thorough examination in cognitive rehabilitation for various conditions, its effectiveness, specifically in TBI, remains limited and inconclusive [20]. Relying solely on rTMS for TBI rehabilitation may offer limited effectiveness compared to a holistic treatment approach that integrates rTMS with other therapies, including pharmacological treatments [21]. However, the available data on using rTMS to supplement pharmaceutical treatments for cognitive rehabilitation is also limited [22].

A promising pharmaceutical treatment for TBI is Cerebrolysin, a medication derived from purified porcine brain proteins. This medication has demonstrated neuroprotective and neurotrophic properties and is believed to facilitate brain cell safeguarding and regeneration. Following an acute brain injury, an inherent, continual brain defense mechanism unfolds in two distinct phases: an immediate response focused on mitigating brain damage (neuroprotection) and a subsequent phase aimed at repairing the inflicted damage (neurorecovery). Within this process, neurotrophic factors emerge as crucial endogenous agents, providing immediate broad-ranging neuroprotective functions and sustaining diverse effects supporting brain resilience and recuperation [23]. Due to this distinctive therapeutic action, treatment with neurotrophic factors relies on recurrent treatment intervals beyond acute administration. Cerebrolysin mimics the actions of neurotrophic factors, targeting four essential natural neurobiological processes: neurotrophicity, neuroprotection, neuroplasticity, and neurogenesis [24].

The CAPTAIN-RTMS trial was the first study to assess the safety and effectiveness of combining rTMS with Cerebrolysin for cognitive rehabilitation in patients with TBI. The primary objectives of this clinical trial were to determine if this combined therapy was feasible, effective, and safe compared to Cerebrolysin alone in improving various neurocognitive outcomes at three and six months post-TBI. Secondary objectives focused on assessing brain electrical activity using electroencephalography and analyzing eye movements with an eye-tracking (ET) device to test if QEEG and ET parameters may be considered possible biomarkers of cognitive dysfunction and neurorehabilitation [25].

## MATERIAL AND METHODS

### Overview

Changes in resting state qEEG activity were investigated in 50 patients from the CAPTAIN-RTMS clinical trial, a phase II, randomized, single-center, double-blind, placebo-controlled research carried out as a component of doctoral studies.

Participants were selected from patients aged 18 and 70 with moderate-to-severe TBI. All patients underwent clinical evaluations and routine treatment upon initial hospital admission. The 180-day study visits and research therapy were conducted on an outpatient basis at the RoNeuro Institute for Neurological Research and Diagnostic in Cluj-Napoca, Romania. Detailed inclusion and exclusion criteria for patients are outlined in the main article [25]. Each participant's study schedule included three visits: Study Day 30 for screening and baseline, Study Day 101 for Visit 1, and Study Day 180 for Visit 2. A third-party provider produced a pre-generated list with random numbers for the patients who matched the study criteria. Three groups were randomly assigned to the participants: (1) Cerebrolysin plus rTMS - CRB+rTMS; (2) Cerebrolysin plus sham rTMS - CRB+SHM; or (3) placebo plus sham rTMS – PLC+SHM. All participants received three treatment cycles. The first group received 30 ml of Cerebrolysin infusions and rTMS sessions for ten days (days 31–40, 61–70, and 91–100). The second group received 30 ml of Cerebrolysin infusions + sham rTMS for the same duration and days as the first group. The third group received a placebo consisting of 250 ml of 0.9% saline solution + sham rTMS. Cerebrolysin was diluted in 0.9% saline solution to a total volume of 250 ml. rTMS was delivered to the left dorsolateral prefrontal cortex (DLPFC) at 10 Hz (10 stimuli/second) with 1,200 stimuli per day for a total of 33 minutes. Stimulation consisted of 40 trains of 3 seconds each, with 20-second intervals between trains.

### EEG recording and analysis

At 30 days and 180 days from the initial TBI, a 5-minute eyes-closed EEG recording was performed using a Nicolet 32-channel Amplifier (Natus). Participants were instructed to remain in a relaxed, wakeful state without undertaking any specific tasks during the recording. EEG data was collected using 32 electrodes (Easycap positioned in accordance with the 10-20 International standard). A band-pass filter with a range of 0.5–40 Hz was applied after the data was sampled at 1024 Hz. Electrode conductance was checked before the recording session to ensure impedances stayed below five kΩ.

EEG data was preprocessed using the BrainVision Analyzer v2.1 software (Brain Products). To remove power line noise, a 60 Hz notch filter was applied to the data after it was down-sampled to 512 Hz. Independent Component Analysis (ICA) with the Informax algorithm was employed to identify and eliminate components related to eye movements, muscle activity, and heartbeat artifacts, ultimately improving data quality. After ICA, any remaining artifacts were manually removed by a trained neurologist, and the data was referenced to the average electrode.

Absolute power spectral density analysis (PSD) was computed in MATLAB R2021a (MathWorks) using the Brainstorm v3.230505 Toolbox [26]. The signals were segmented into 4-second intervals with a 50% overlap, employing Welch’s method to assess the power distribution of EEG data across various frequencies. Subsequently, the Discrete Fourier Transform was applied to each window, and the squared magnitude of the Fourier coefficients was averaged across all segments. The power spectrum was defined within distinct frequency bands - delta 0-4 Hz, theta 4-8 Hz, alpha 8-13 Hz, and beta 13-30 Hz. We computed the following PSD ratios utilizing the absolute PSD values: DAR, DTABR, and TBR. Mean values of PSD ratios were calculated for the entire cortex/brain/scalp - Fp1, Fp2, F3, F4, F7, F8, Fz, C3, C4, Cz, P3, P4, P7, P8, Pz, O1, O2, Oz, FC1, FC2, FC5, FC6, CP1, CP2, CP5, CP6, T7, T8, TP9, TP10, PO9 and PO10, and for the frontal region - Fp1, Fp2, F3, F4, FC5, FC6, FC1, FC2 and Fz.

### Statistical analysis

Comparisons of qEEG indexes were performed within treatment groups between visits using the paired Wilcoxon signed-rank test. Comparisons between groups at each visit were done using the Wilcoxon rank-sum test (also known as the Mann-Whitney-U test). Cliff’s Delta [27] and its paired equivalent were chosen effect size measures for this study because they can be applied without the assumption of normally distributed data.


$$\delta \text{ =}\frac{\sum sign(d_{i,j})}{i\;*j}\;\delta_{paired}=\frac{\sum sign(Y_{i}-X_{i})}{n}$$

Statistical analysis was conducted using R v4.3.1 [28], with libraries: igraph [29], MANOVA.RM [30], R.matlab [31], ggplot2 [32], openxlsx [33], and patchwork [34]. The statistical analysis was conducted with QSAP, an R-based platform designed to examine quantitative electroencephalography characteristics and their association with clinical information [35].

## RESULTS

### Delta alpha ratio (DAR)

**Figure 1:**
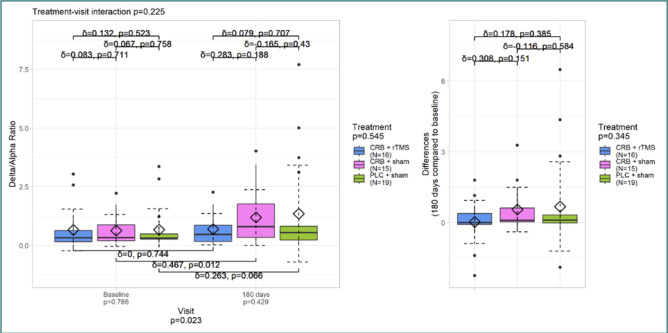
DAR – all electrodes

**Figure 2:**
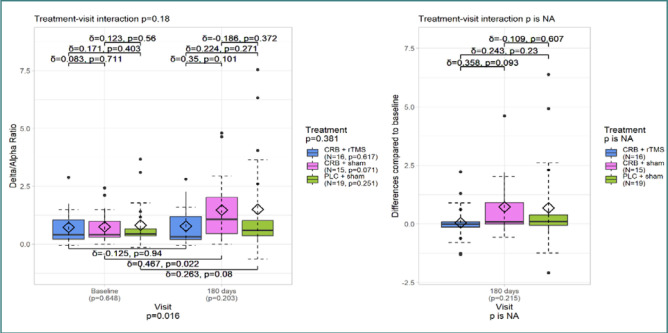
DAR – frontal electrodes

Figures 1 and 2 illustrate the differences in DAR between the initial and last visits. Figure 1 displays the DAR calculated for all electrodes, while Figure 2 focuses specifically on the frontal electrodes.

There was a significant increase in DAR values (*P* = 0.023) from the initial visit to the last visit (Figure 1). However, this change does not appear to be due to the therapeutic effect (*P* = 0.225). When comparing treatment groups, the Cerebrolysin and sham groups showed the largest effect size (*P* <0.01; δ =0.46). There were no additional differences between the groups or visits.

Similar results were observed for frontal DAR (Figure 2), with a significant change only between visits (*P* = 0.016). However, the CRB + sham treatment group, with the largest effect size (δ = 0,467), also had a significant *P* value.

### Delta and theta to alpha and beta ratio (DTABR)

**Figure 3:**
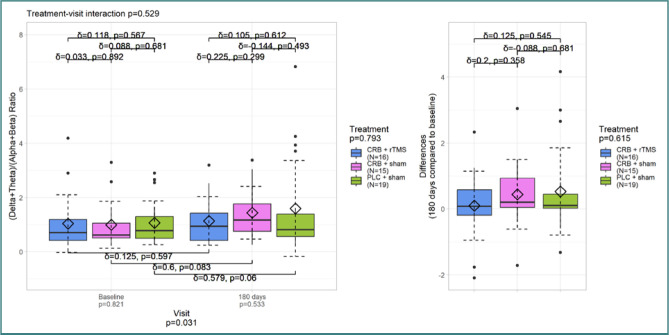
DTABR – all electrodes

Figure 3 illustrates the variations in DTABR values, demonstrating a significant increase in DTABR at the last visit (*P* value between visits 0.031). There was no evidence of any therapeutic impact. Although not statistically significant, patients receiving Cerebrolysin and sham treatment had a medium effect size (δ = 0,6), while the PLC + SHAM group had a slightly lower value.

**Figure 4:**
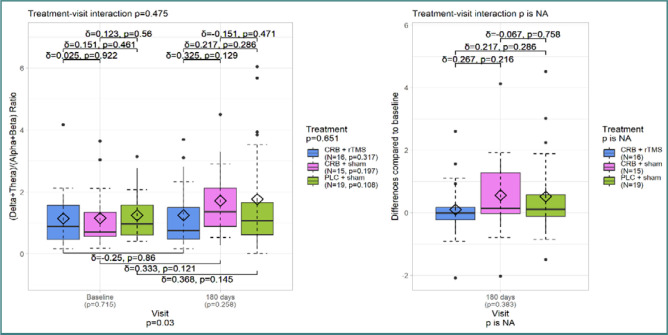
DTABR – frontal electrodes

Similar results were observed for DTABR values from frontal electrodes (Figure 4), with a significant increase at the third visit (*P* < 0.03). There were no additional significant findings, and the effect size between the treatment groups was modest.

### Theta beta ratio (TBR)

**Figure 5:**
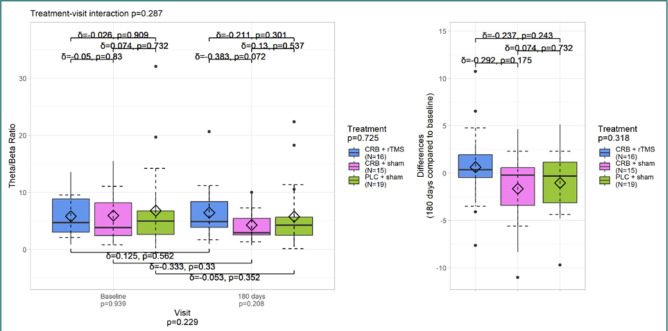
TBR – all electrodes

**Figure 6:**
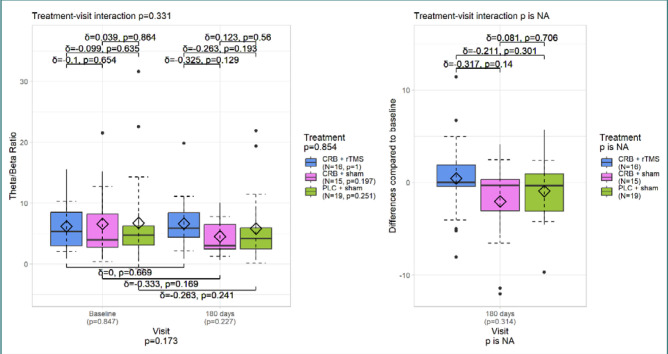
TBR – frontal electrodes

The analysis of TBR for all electrodes (Figure 5) showed that its values were slightly lower at the last visit but with a non-significant *P* value of 0.229. There was no significant difference in the treatment-visit interaction. However, the Cerebrolysin + sham treatment group had a moderate effect size. Similar results were observed for the frontal electrodes (Figure 6).

## DISCUSSION

This secondary analysis of the CAPTAIN-RTMS clinical trial is the first to investigate the combined efficacy of Cerebrolysin and rTMS for treating TBI, comparing it to Cerebrolysin monotherapy or placebo. The multi-arm design of the study aimed to identify the most effective intervention while allowing for the comparison of individual effects. While the primary objectives included the efficacy of the treatment on neurocognitive tests, besides the feasibility and safety of this approach, for the secondary objectives of the study, we chose to analyze the evolution of eye movements and brain electrical activity using an eye-tracking device and quantitative electroencephalography.

The majority of individuals with TBI exhibit abnormal EEG patterns. During the acute stage, epileptiform activity characterized by high-amplitude sharp waves or high-frequency discharges can be found, followed by diffuse slowing on the EEG with elevated theta and delta, an elevated theta/alpha ratio, and decreased alpha frequency. Most abnormalities in EEGs following head trauma resolve within a year, with the bulk of them disappearing within the first three months. However, in people with chronic cognitive or psychiatric symptoms connected to the TBI, a higher power in slow waves and a lower power in the alpha band can be persistent for more than one year after the injury [36].

Our qEEG analysis focused on three specific ratios –DAR, DTABR, and TBR – which reflect the balance between slow and fast brainwave activity. These parameters were chosen because DAR values have been reported as the most consistent neural feature compared to analyzing individual frequency bands, showing the best correlation with the degree of brain recovery. Additionally, DTABR, encompassing all four types of frequency bands, has been considered an effective predictor of neurological changes [6].

Several studies correlated qEEG ratios with cognitive functions, emotions, and stress. Rhee *et al*. [37] associated lower DAR values with lower mental workloads and diminished cognitive stress, while reduced TBR indicated increased attention. Conversely, high values of TBR were associated with cognitive dysfunction, memory and executive dysfunction, and an increased risk of hallucinations in patients with Lewy Body Disease [38]. Gazzellini *et al*. suggested that TBR could be a suitable electrophysiological marker of brain dysfunction and attention lapses in patients with TBI, mainly those with frontal lobe lesions [39].

In a study on patients diagnosed with acute ischemic stroke, Bentes *et al*. [12] used qEEG indexes to predict outcomes. DTABR showed the highest discriminative capacity, and the superior values of DTABR predicted a poor functional outcome at 12 months after stroke, as measured by the modified Rankin scale. In a similar study, Schleiger *et al*. [7] demonstrated that increased DAR was negatively correlated with functional and cognitive outcomes after stroke. Leon-Carrion *et al*. [9] also showed a strong negative correlation between DAR values and functional outcomes six months after neurorehabilitation in patients with TBI [9].

Several studies have explored the impact of Cerebrolysin on brain bioelectrical activity, consistently showing a reduction in slow EEG activity and an increase in faster frequencies. Alvarez *et al*. [40,41] found significant cognitive improvement and accelerated reduction of qEEG slowing in patients with TBI receiving Cerebrolysin. Similar outcomes were reported by Muresanu *et al*. with patients with mild to moderately probable vascular dementia. The worst cognitive performance was associated with increased EEG slowing, respectively, with higher power in delta and theta frequencies and reduced alpha power. However, treatment with Cerebrolysin improved cognitive performance and diminished EEG slowing for at least 12 weeks after the therapy, indicating a positive connection between changes in cognition and qEEG activity induced by CRB [40,41].

In contrast, the 10 Hz repetitive transcranial magnetic stimulation induced an overall increase in delta power in healthy subjects and also in patients with major depressive disorder. However, the qEEG recording was performed after the rTMS sessions, and it is not determined how long these qEEG alterations were maintained [42,43].

The results of this study are different from those of previous literature. Regardless of the type of treatment, all patients had an increase in delta power at 180 days from the TBI onset compared to baseline, both for frontal and all electrodes. This finding was reflected in the significant increase of DAR and DTABR values for all patients without any change in the treatment-visit interaction. The largest effect sizes were observed in the Cerebrolysin + sham group for both DAR and DTABR values, particularly for frontal DAR. This may suggest that rTMS did not have any influence on the cerebral electrophysiological changes, while the CRB treatment was an important factor. Despite the previously reported correlation between increased DAR/DTABR and poorer functional and cognitive outcomes [40,41,44,45], our qEEG results correlated with improved, non-significant results at the neurocognitive tests for the CRB + TMS and CRB + sham groups [25].

TBR values were contrasting, being slightly lower at the third visit compared to the first evaluation but without any statistical significance. The reduction of theta power may correlate with improved cognitive rehabilitation, mainly with increased attention [37].

Nevertheless, we must also take into consideration the limitations of our study. The relatively small sample size, with patients from a single site and lacking severity stratification, may limit the generalizability of our findings to a larger population. Also, the duration of the study was relatively short, and the neurorehabilitation process of patients with TBI is often lengthy, so long-term follow-up evaluations should always be considered.

## CONCLUSION

The CAPTAIN-RTMS trial offered valuable insights into the potential role of qEEG parameters in monitoring and understanding the recovery trajectory in patients after TBI. By evaluating indices like DAR, DTABR, and TBR, the study contributed to the complex understanding of brain functions post-TBI. Although the trial presents compelling evidence, the inherent limitations highlight the necessity for further research. Larger-scale studies with more diverse populations and extended follow-ups are essential to validate these findings and to refine therapeutic strategies.

## Data Availability

Further data is available from the corresponding author on reasonable request.
